# Ubiquitin conjugation to Gag is essential for ESCRT-mediated HIV-1 budding

**DOI:** 10.1186/1742-4690-10-S1-O5

**Published:** 2013-09-19

**Authors:** Paola Sette, Kunio Nagashima, Robert Piper, Fadila Bouamr

**Affiliations:** 1 Infectious Diseases, National Institutes of Health, Bethesda, MD, USA; 2 Image Analysis Laboratory, Advanced Technology Program, SAIC-Frederick, NCI-Frederick, Bethesda, MD, USA; 3 Molecular Physiology and Biophysics, University of Iowa, Iowa City, Iowa, USA

## 

HIV-1 relies on the host ESCRTs for release from cells. HIV-1 Gag engages ESCRTs by directly binding TSG101 or Alix. ESCRTs also sort ubiquitinated membrane proteins through endosomes to facilitate their lysosomal degradation. The ability of ESCRTs to recognize and process ubiquitinated proteins suggests that ESCRT-dependent viral release may also be controlled by ubiquitination. Although both Gag and ESCRTs undergo some level of ubiquitination, definitive demonstration that ubiquitin is required for viral release is lacking. Here we suppress ubiquitination at viral budding sites by fusing the catalytic domain of the Herpes Simplex UL36 deubiquitinating enzyme (DUb) onto TSG101, Alix, or Gag.

Expressing DUb-TSG101 suppressed Alix-independent HIV-1 release and viral particles remained tethered to the cell surface. DUb-TSG101 had no effect on budding of MoMLV or EIAV, two retroviruses that rely on the ESCRT machinery for exit. Alix-dependent virus release such as EIAV’s, and HIV-1 lacking access to TSG101, was instead dramatically blocked by co-expressing DUb-Alix. Finally, Gag-DUb was unable to support virus release and dominantly interfered with release of wild type HIV-1. Fusion of UL36 did not affect interactions with Alix, TSG101, or Gag and all of the inhibitory effects of UL36 fusion were abolished when its catalytic activity was ablated. Accordingly, Alix, TSG101 and Gag fused to inactive UL36 functionally replaced their unfused counterparts. Interestingly, coexpression of the Nedd4-2s ubiquitin ligase suppressed the ability of DUb-TSG101 to inhibit HIV-1 release while also restoring detectable Gag ubiquitination at the membrane. Similarly, incorporation of Gag-Ub fusion proteins into virions lifted DUb-ESCRT inhibitory effect. In contrast, Nedd4-2s did not suppress the inhibition mediated by Gag-DUb despite restoring robust ubiquitination of TSG101/ESCRT-I at virus budding sites.

These studies provide the first demonstration of a necessary and natural role for ubiquitin in ESCRT-dependent viral release and indicate a critical role for ubiquitination of Gag rather than ubiquitination of ESCRTs themselves.

